# Comparison of Zidovudine and Tenofovir Based Regimens With Regard to Health-Related Quality of Life and Prevalence of Symptoms in HIV Patients in a Kenyan Referral Hospital

**DOI:** 10.3389/fphar.2018.00984

**Published:** 2018-10-12

**Authors:** Jilian O. Etenyi, Faith A. Okalebo, Margaret Oluka, Kipruto A. Sinei, George O. Osanjo, Amanj Kurdi, Johanna C. Meyer, Brian Godman, Sylvia Opanga

**Affiliations:** ^1^Department of Pharmacology and Pharmacognosy, School of Pharmacy, University of Nairobi, Nairobi, Kenya; ^2^Department of Pharmaceutics and Pharmacy Practice, School of Pharmacy, University of Nairobi, Nairobi, Kenya; ^3^Strathclyde Institute of Pharmacy and Biomedical Sciences, University of Strathclyde, Glasgow, United Kingdom; ^4^Department of Pharmacology, College of Pharmacy, Hawler Medical University, Erbil, Iraq; ^5^Department of Public Health Pharmacy and Management, School of Pharmacy, Sefako Makgatho Health Sciences University, Pretoria, South Africa; ^6^Department of Laboratory Medicine, Division of Clinical Pharmacology, Karolinska Institutet, Solna, Sweden; ^7^Health Economics Centre, Management School, University of Liverpool, Liverpool, United Kingdom

**Keywords:** HRQoL, MOS-HIV, side-effects, tenofovir, zidovudine, antiretroviral treatments, Kenya

## Abstract

**Aim:** Zidovudine and tenofovir form the backbone of antiretroviral therapy in Kenya. However, their side-effects may affect the quality of life (QoL) of patients. The aim was to compare the health-related quality of life (HRQoL) of adult patients on tenofovir versus zidovudine based regimens in a referral hospital in Kenya to provide future guidance.

**Methods:** A comparative cross sectional study among 501 adult out-patients on either tenofovir or zidovudine was undertaken in Kenyatta National Hospital between 2015 and 2016. The Medical Outcome Study HIV Health Survey (MOS-HIV) was administered along with other key aspects of treatment. Linear regression analysis was performed to identify determinants of HRQoL.

**Results:** Patients on zidovudine had a higher Physical Health Summary Score (PHSS) and Mental Health Summary Score (MHSS) compared to those on tenofovir. The presence of any symptom of the disease and a stated inability to cope were negatively associated with PHSS, whilst having a regular source of income improved PHSS. Being on tenofovir, symptom of illness [β = -1.24; 95% CI (-2.253, -0.226)], absence of pain [β=0.413; 95% CI (0.152, 0.674)] and patient stated inability to cope with HIV [β = -1.029; 95% CI (-1.441, -0.617)] affected the MHSS. Patients on tenofovir and second line regimens had more signs and symptoms of illness.

**Conclusion:** Participants on zidovudine based regimens showed a better performance across all aspects of HRQoL. These are considerations for the future.

## Introduction

The World Health Organization defines health as “*a state of complete physical, mental and social well-being and not merely the absence of disease or infirmity*” (WHO Constitution of The World Health Organization, 2006). Diseases affect the psycho-social status of an individual by increasing the economic burden, psychological distress, and social unacceptability of patients especially with conditions associated with stigma, such as Human Immunodeficiency Virus (HIV) infection. This non-physical effect of HIV, and the associated medicines, can lead to despair and even non-adherence to treatment (Campos et al., 2009; National AIDS STI Control Programme [NASCOP], 2011). Consequently, there is a need for studies to evaluate the impact of treatments on health-related quality of life (HRQoL) especially in diseases such as HIV associated with stigma, where there are also concerns with the toxicity of treatments, and in HIV patients with combined morbidities, to guide future strategies (Oguntibeju, 2012; Balderson et al., 2013; Degroote et al., 2014; Vagiri et al., 2014, 2015; Rogers, 2015; Mafirakureva et al., 2016; Brandt et al., 2017).

HIV/AIDS is prevalent in sub-Sahara Africa, accounting for up to 80% of the world’s HIV infected population, and is a leading cause of death and morbidity in these countries (National AIDS STI Control Programme [NASCOP], 2011; Rogers, 2015). For instance, in Botswana at one stage nearly 50% of women aged between 30 and 34 years had HIV, which has potential implications for quality of life (QoL) and adherence to treatments if patients also have concomitant non-communicable diseases (Rwegerera et al., 2018). This reflects the greater prevalence of HIV among women than men in sub-Sahara Africa, different to the populations typically seen in Western countries (Gaida et al., 2016; Kalemeera et al., 2016; Ayele et al., 2017a; Mataranyika et al., 2018). In Kenya, the incidence rate for new HIV infections was recently estimated at 137,200 with an estimated 1.88 million patients living with HIV (Wang et al., 2016).

HIV is highly stigmatized to the extent that it may lead to delayed health seeking behavior, access to treatment and in some instances suicide (Lieber et al., 2006; Maimaiti et al., 2017; Cornelius et al., 2018; Wang et al., 2018). Having said this, access to HIV treatment has appreciably improved in Eastern and Southern Africa in recent years (Dadi et al., 2017). Opportunistic infections associated with HIV/AIDS may also cause devastating psychological distress and decrease the ability of patients to perform normal duties (Wang et al., 2018). In addition, antiretroviral (ARV) medicines can appreciably reduce morbidity and mortality, including significant physical improvement and associated HRQoL. However, these medicines may also cause severe side-effects themselves potentially impacting on any improvement in HRQoL that could be gained through treatment, though some regimens are becoming more tolerable (Burgoyne and Tan, 2008; Mannheimer et al., 2008; Campos et al., 2009; Oguntibeju, 2012; Velen et al., 2013; Dadi et al., 2017; Pasquau et al., 2018).

Tenofovir is a nucleotide reverse transcriptase inhibitor (NtRTI), an ARV that is commonly used in combination with other ARVs in the management of HIV infection in Kenya and elsewhere as part of the first line regimen (National AIDS STI Control Programme [NASCOP], 2011). However, it is associated with side-effects (Subbaraman et al., 2007; Nemaura et al., 2013; Waheed et al., 2015). Zidovudine is an ARV which belongs to the class of nucleoside reverse transcriptase inhibitors (NRTIs) (Velen et al., 2013; Dadi et al., 2017; Ayele et al., 2017a). Both are widely used in Kenya in combination for the management of patients with HIV/AIDS (National AIDS STI Control Programme [NASCOP], 2011).

Zidovudine is used as an alternative to tenofovir in antiretroviral treatment (ART) regimens, although it is also associated with side-effects such as chronic anemia, which are likely to affect patients’ QoL. Tenofovir is claimed to have a better safety profile compared to zidovudine, with the main side-effect being renal toxicity (Awino, 2008; Dadi et al., 2017). Some studies have shown that tenofovir is superior to zidovudine in terms of its safety profile because of a reduced incidence of anemia and fat redistribution (Wandeler et al., 2014; Insaniputri et al., 2017). Other studies have also shown that tenofovir had fewer side-effects, but the range of side-effects studied was limited (Saez-Llorens et al., 2015). However, this still remains to be seen across populations in sub-Sahara Africa.

Currently few studies have been conducted in Kenya on the HRQoL of people living with HIV who are on ART. This is very important given the appreciable number of citizens with HIV in Kenya and concerns with accessing and adhering to treatment in reality (Wang et al., 2016). Consequently, this study aimed to compare the HRQoL of adult HIV positive patients on tenofovir versus zidovudine based regimens and to determine potential factors that affect HRQoL. In addition, to measure the prevalence and risk factors for the presence of symptoms of illness or side-effects in HIV patients. The findings will provide evidence to influence future treatment selection and policy decisions within Kenya and wider.

## Materials and Methods

### Design, Study Site, and Population

A comparative cross sectional study was undertaken using the Medical Outcome Study HIV Health Survey (MOS-HIV) questionnaire (Wu et al., 1997). The study was conducted at Kenyatta National Hospital (KNH) Comprehensive Care Center (CCC). KNH is the main referral and teaching hospital in Kenya for patients with HIV, and manages approximately 5,000 of these patients. Consequently, KNH CCC could provide comprehensive data for assessing these issues. The study population was adult HIV positive patients attending the CCC clinic at KNH between December 2015 and May 2016. The patients were either on tenofovir or zidovudine based regimens. The current Kenyan guidelines for treatment of HIV (National AIDS STI Control Programme [NASCOP], 2011) recommend the use of tenofovir, lamivudine, and efavirenz (TDF+3TC+EFV) as first line therapy among patients older than 15 years. Previous guidelines recommended the use of a zidovudine based regimen as first line therapy.

### Sample Size Considerations and Sampling

The Lui’s (1991) formula was used for sample size computation, as this was a two arm comparative study with a continuous outcome variable. The population variance of HRQoL was assumed to be 22.5, as obtained from a study conducted by Mast et al. (2004). The difference in the HRQoL between the two groups was assumed to be 2, the level of significance was set at 0.05 and therefore the standard normal deviate used in the formula was 1.96. The power of the study (1-β) was set at 90%, hence the conventional multiplier for power was 1.282. Using this formula, the calculated minimal sample size was 217 per arm. This figure was inflated by 10% to compensate for non-responses.

The final calculated sample size was 238 per arm (Lui, 1991):

$$\begin{array}{c} 2N=[{4(Z\alpha /2+Z}_{1-\beta }{)}^{2}\sigma^{2}{]/\delta }^{2} \end{array}$$

Where:

*Z*α/2 = the standard normal deviate at 95% CI (1.96)

*Z*_1-β_ = the *Z*-value corresponding to a power of 90 (1.282)

### Eligibility Criteria and Participant Recruitment

Adult patients of either sex who gave informed consent, and who had been on either tenofovir or zidovudine based regimens for at least 6 months, were included in the study. Patients were recruited during their clinic days. Those who consented were recruited within the pharmacy as they were collecting their medicines.

A total of 6 months was chosen for the inclusion criteria because longitudinal studies have shown significant changes in QoL of patients who started on ART after this period, regardless of the CD4^+^ count (Subbaraman et al., 2007). For this reason, patients who had been on therapy for less than 6 months were deliberately excluded. Individuals who were expectant mothers or those who did not meet the inclusion criteria were also excluded.

### Data Collection Tool, Patient Interview, and Abstraction of Patient Records

The MOS-HIV questionnaire was used to collect data on HRQoL since unlike other tools for assessing HRQoL in HIV patients, it was specifically designed to collect information on patient reported symptoms in HIV positive patients. In addition, the physical and mental health aspects of HRQoL are separately measured using this tool. It has also been extensively used in a wide variety of settings with consistent reliability and validity (Mast et al., 2004), and has been validated in a study in Uganda, which has a similar cultural background as Kenya. In the Ugandan study, the tool was found to have good construct- and face validity and reliability (Mast et al., 2004).

Research assistants together with the principal investigator collected the data. The research assistants were trained nurses who worked within the Comprehensive Care Clinic where the data was collected. Patients report to the clinic every 3 months. Approximately 10 patients were seen daily during the study period. To ensure confidentiality, only one participant was interviewed at a time in the pharmacy consultation room before they collected their medicines.

The MOS-HIV questionnaire was administered to participants during the interview. Additional information on socio-demographic characteristics, symptoms participants were experiencing, satisfaction with service provision, coping mechanisms, adherence to medication, and alcoholism/substance dependence were also obtained during the interview using additional questions that were not part of the MOS-HIV questionnaire (Wu et al., 1997; Mast et al., 2004). Data on the recent CD4^+^ count and viral load was extracted from the patients’ records.

### Computation of the Scores of Individual Domains of Health

The raw scores of each of the domains were computed as described in the MOS-HIV Health Survey User’s Manual (Wu et al., 1991, 1997). All the items and scales were scored so that a higher score indicated better health. 11 items were reverse coded because a higher pre-coded item value indicated a poorer health state. This ensured that a higher value indicated better health on all MOS-HIV items and scales.

Each of the questions entailed responses that had a value which ranged from 1 to 2, 1 to 4, and 1 to 6, based on the Likert scale. The sum of the scores of the responses from the questions in a particular domain were taken as the raw score.

The scores were subsequently transformed to a scale of 0–100. Higher score values represented a better HRQoL while a lower score indicated poorer HRQoL. The method for transformation of the scores is described in the MOS questionnaire user’s manual (Wu et al., 1991).

### Computation of the Physical- and the Mental Health Summary Score

The scores for each domain were used to obtain both the Physical Health Summary Scores (PHSS) and the Mental Health Summary Scores (MHSS). *Z*-score transformation for standardization of the scores to the standard Roche patient population was undertaken as described in the MOS questionnaire user’s manual (Wu et al., 1991). The Roche patient population was a group of HIV positive patients on whom validation of the MOS-HIV tool was performed (Wu et al., 1997).

The obtained PHSS and MHSS were subsequently transformed to have a mean of 50 and a SD of 10 in order to obtain the final PHSS and MHSS as described in the MOS-HIV manual (Wu et al., 1997).

### Data Management and Quality Assurance

Data was entered in an Epi Info Version 7 database within 24 h after completion of the questionnaire. Evaluation for completeness and accuracy was undertaken daily and data backed-up weekly. Confidentiality was maintained by using patient codes instead of identifier information. Training and explanation of the nature of the study was undertaken with the research assistants prior to the study. Pre-testing of the questionnaire was undertaken on 20 patients with subsequent refinement of the actual questionnaire. This only related to the data collection section on socio-demographic characteristics, CD4^+^ counts, and patient-related symptoms.

### Variables and Statistical Analysis

The three dependent variables were PHSS, MHSS and the prevalence of symptoms of disease/side-effects. Independent variables were ARV regimens, socio-demographic traits, and HIV-related history.

Descriptive statistics were used and the Shapiro–Wilk test performed to test whether continuous variables were normally distributed. They were summarized using the median and the interquartile range. Categorical variables were summarized as counts and percentages.

Distribution of all variables was compared across the two groups using inferential tests which included Chi-square, Mann–Whitney, and un-paired Student’s *t*-tests. Logistic regression analysis was used to identify the risk factors for any symptoms of disease or side-effects. Linear regression with robust estimation was conducted to identify predictors of the MHSS and PHSS. Model building was undertaken using a forward stepwise approach. The level of significance was set at 0.05. STATA version 10.0 was used for data analysis.

### Ethical Considerations

Ethical approval was obtained from Kenyatta National Hospital/University of Nairobi (KNH/UoN) Ethics and Research Committee (Approval Reference Number: KNH-ERC/A/467). Institutional approval was sought from KNH. Informed consent was obtained from patients. Privacy was maintained by conducting the interview in a secluded room. Confidentiality was maintained by using codes instead of patient identifier information.

## Results

### Participants’ Baseline Socio-Demographic Characteristics

A total of 501 patients were recruited. Most (60.8%) of the patients were on tenofovir based regimens. Most were female (348; 69.5%) and were aged between 36 and 45 years. Less than half of the participants (234; 46.7%) had attained secondary level education and approximately half were married. Most patients (77.8%) had disclosed their HIV status to at least a friend or relative. There were no statistically significant differences in the socio-demographic traits across the two arms (**Table 1**).

**Table 1:** Baseline socio-demographic characteristics of study participants (*n* = 501).

Variable	Tenofovir (*n* = 301)	Zidovudine (*n* = 200)	Total (*n* = 501)	*p*-Value
**Age (years)**				
18–35	66 (21.9)	50 (25)	116 (23.2)	
36–45	127 (42.2)	86 (43)	213 (42.5)	
46–64	104 (34.6)	62 (31)	166 (33.1)	0.809
≥ 65	3 (0.9)	2 (1)	5 (0.9)	
Missing	1 (0.3)	–	1 (0.2)	
**Sex *n* = 501**				
Male	87 (28.9)	63 (31.5)	150 (29.9)	
Female	212 (70.4)	136 (68)	348 (69.5)	0.619
Missing	2 (0.7)	1 (0.5)	3 (0.59)	
**Marital status**				
Married	158 (52.5)	106 (53)	264 (52.6)	
Married/Cohabiting	3 (0.9)	5 (2.5)	8 (1.6)	0.332
Single	61 (20.3)	33 (16.5)	94 (18.8)	
Widowed	46 (15.3)	26 (13)	72 (14.4)	
Divorced	33 (10.9)	29 (14.5)	62 (12.4)	
Missing	–	1 (0.5)	1 (0.2)	
**Regions**				
Nyanza	75 (24.9)	36 (18)	111 (22.2)	
Western	49 (16.3)	28 (14)	77 (15.4)	
Rift valley	13 (4.3)	12 (6)	25 (4.9)	
Nairobi	6 (1.9)	4 (2)	10 (1.9)	0.537
Central	89 (29.6)	62 (31)	151 (30.1)	
Eastern North	55 (18.3)	48 (24)	103 (20.5)	
Eastern	5 (1.7)	3 (1.5)	8 (1.6)	
Coast	5 (1.7)	4 (2)	9 (1.8)	
Missing	4 (1.3)	3 (1.5)	7 (1.4)	
**HIV status disclosure**				
Yes	232 (77.1)	158 (79)	390 (77.8)	0.433
No	67 (22.3)	38 (19)	105 (20.9)	
Missing	2 (0.4)	4 (2)	6 (1.2)	
**Educational level**				
No education	7 (2.3)	2 (1)	9 (1.8)	
Primary	53 (17.6)	48 (24)	101 (20.2)	
Secondary	144 (47.8)	90 (45)	234 (46.7)	
Tertiary/University	95 (31.6)	55 (27.5)	150 (29.9)	0.121
Others	0 (0)	2 (1)	2 (0.4)	
Missing	2 (0.7)	3 (1.5)	5 (0.9)	
**Religious belief**				
No	4 (1.3)	1 (0.5)	5 (0.9)	
Yes	294 (97.7)	197 (98.5)	491 (98)	0.653
Missing	3 (0.9)	2 (1)	5 (0.9)	
**Regular source of income**				
No	66 (21.9)	38 (19)	104 (20.8)	0.572
Yes	232 (77.1)	155 (77.5)	387 (77.3)	
Missing	3 (0.9)	7 (3.5)	10 (1.9)	

### Baseline Medical Characteristics of Study Participants

There was a statistically significant difference in the duration of HIV infection and the duration on ART (*p* < 0.001) across the two arms. Those patients on zidovudine had been on treatment longer as zidovudine was the first line regimen before tenofovir was introduced. A total of 47.3% had been HIV positive for 5.1 to 10 years and 44.7% had a CD4^+^ count of > 500 cells/mm^3^ and above.

Out of the participants who had a viral load report, 67.7% had a viral load of less than 0.01 copies/ml. 21.6% of participants in the tenofovir arm had been on therapy for 2 years and below compared to only 5% in the zidovudine arm. In the tenofovir arm, 11% of the participants had been on treatment for more than 10 years compared to only 8% in the zidovudine arm. The CD4^+^ counts and adherence were comparable across the arms. The baseline medical characteristics of study participants are presented in **Table 2**.

**Table 2:** Baseline medical history of participants on tenofovir and zidovudine based regimens (*n* = 501).

Variable	Tenofovir (*n* = 301)	Zidovudine (*n* = 200)	Total (*n* = 501)	*p*-Value
**Duration of HIV infection in years**				
≤2.0	39 (13)	5 (2.5)	44 (8.8)	
2.1–5.0	70 (23.3)	46 (23)	116 (23.2)	
5.1–10.0	124 (41.2)	113 (56.5)	237 (47.3)	
10.1–15.0	49 (16.3)	25 (12.5)	74 (14.8)	**<0.001**
>15	19 (6.3)	10 (5)	29 (5.8)	
Missing	–	1 (0.5)	1 (1.2)	
**Duration on ART in years**				
≤1.0	35 (11.6)	3 (1.5)	38 (7.6)	
1.1–2.0	30 (10)	7 (3.5)	37 (7.4)	
2.1–5.0	82 (27.2)	62 (31)	144 (28.7)	
5.1–10	121 (40.2)	111 (55.5)	232 (46.3)	**<0.001**
>10	33 (11)	16 (8.0)	49 (9.8)	
Missing	–	1 (0.5)	1 (0.2)	
**Adherence: Missed drug intake**				
Missing	6 (2.0)	1 (0.5)	7 (1.4)	
No	195 (64.8)	142 (71)	337 (67.3)	0.187
Yes	100 (33.2)	57 (28.5)	157 (31.3)	
**Recent CD4^+^ count in cells/mm^3^**				
<250	41 (13.6)	37(18.5)	78 (15.6)	
250–499	108 (35.9)	71 (35.5)	179 (35.7)	
500–999	127 (42.2)	73 (36.5)	200 (39.9)	0.414
≥1000	15 (4.9)	9 (4.5)	24 (4.8)	
Missing	10 (3.3)	10 (5)	20 (3.9)	
**Recent viral load copies/ml *n* = 158**				
0.01	62 (70.5)	45 (64.3)	107 (67.7)	
1–1000	14 (15.9)	7 (10)	21 (13.3)	0.121
>1000	12 (13.6)	18 (25.7)	30 (19)	

*NB: Bold under *p*-value denotes statistical significance.*

### Antiretroviral Treatment Regimens of the Participants

The majority of the participants were on a first line regimen compared to only 9.4% on a second line regimen. Most of the participants were on TDF/3TC/EFV (43.5%). The ratio of participants on tenofovir to zidovudine was 6:4 (**Table 3**).

**Table 3:** ART regimens of study participants at Kenyatta National Hospital (*n* = 501).

Variable	AZT-based regimens (*n*, %)		TDF-based regimens (*n*, %)		Total (*n*, %)
**Type of ART regimen**					
	AZT/3TC/NVP	89 (17.8)	TDF/3TC/NVP	65 (12.9)	
	AZT/3TC/EFV	82 (16.4)	TDF/3TC/EFV	218 (43.5)	
	AZT/3TC/LPV/r	21 (4.2)	TDF/3TC/LPV/r	17 (3.4)	
	AZT/3TC/ATV/r	8 (1.6)	TDF/3TC/ATV/r	1 (0.2)	
	**Total**	200 (39.9)	**Total**	301 (60.1)	
**Participants on selected drugs (*n*, %)**					
EFV		82 (41)		218 (72.4)	300 (59.9)
NVP		89 (44.5)		65 (21.6)	154 (30.7)
LPV/r		21 (10.5)		17 (5.7)	38 (7.6)
ATV/r		8 (4)		1 (0.3)	9 (1.8)
**Total**		200 (100)		301 (100)	501 (10.0)
First line therapy		171 (85.5)		283 (94.0)	454 (90.6)
Second line therapy		29 (14.5)		18 (6.0)	47 (9.4)

*NB: TDF-Tenofovir, 3TC-Lamivudine, EFV-Efavirenz, NVP-Nevirapine, LPV/r-Lopinavir/ritonavir, ATV/r-Atazanavir/ritonavir, DRV/r-Duranavir/ritonavir, AZT-zidovudine.*

All the study participants were on lamivudine, irrespective of whether they were on tenofovir or zidovudine. Out of 47 patients on second line therapy, the majority (29) were on zidovudine while 18 were on tenofovir. Most patients on tenofovir based regimens were on efavirenz as opposed to the zidovudine based arm where most were on nevirapine.

### Coping Mechanisms and Satisfaction With Services Provided

Nearly a third of the participants embraced sharing about their illness as a coping style (35.3%). The majority of the participants were very satisfied with the quality of service offered by the health care providers. Nearly, a third (28.1%) of participants took alcohol to help with coping.

There was a significant difference (*p* = 0.034) in the type of coping mechanisms across the regimens. Participants on tenofovir had accepted their HIV status while those on zidovudine adopted sharing about their illness to help them to cope. When compared to the tenofovir arm, slightly more participants on zidovudine had joined a support group. The coping mechanisms adopted are summarized in **Table 4**.

**Table 4:** Coping mechanisms of the study participants and satisfaction with service provided.

Variable	Tenofovir (*n* = 301)	Zidovudine (*n* = 200)	Total (*n* = 501)	*p*-Value
**Coping strategy**				
Acceptance	104 (34.6)	50 (25)	154 (30.7)	**0.034**
Sharing about illness	93 (30.8)	84 (42)	177 (35.3)	
Support group	87 (28.9)	60 (30)	147 (29.3)	
Spiritual support	13 (4.3)	6 (3)	19 (3.8)	
Any other	3 (0.9)	0 (0)	3 (0.6)	
Missing	1 (0.3)	–	1 (0.2)	
**Satisfaction with service provision**				
Very good	195 (64.8)	144 (72)	339 (67.7)	
Good	96 (31.9)	47 (23.5)	143 (28.5)	0.138
Fair	8 (2.7)	8 (4)	16 (3.2)	
Bad	1 (0.3)	1 (0.5)	2 (0.4)	
Missing	1 (0.3)	–	1 (0.2)	
**Alcoholism/substance dependence**				
Use alcohol	87 (28.9)	54 (27)	141 (28.1)	
Hard drugs	5 (1.7)	5 (2.5)	10 (1.9)	0.704
No drugs	202 (67.1)	139 (69.5)	341 (68.1)	
Missing	7 (2.3)	2 (1)	9 (1.8)	

*NB: Bold under *p*-value denotes statistical significance.*

### Prevalence of Adverse Symptoms of Disease and Side-Effects

The overall prevalence of any adverse signs and symptoms including side-effects was 15.5%. Five (1%) of the participants did not give a response. There was a statistically significant difference in the prevalence of adverse symptoms and side-effects across the two arms (*p* = 0.005).

Out of the participants who had symptoms and side-effects that compromised their HRQoL, 57 (74.0%) patients were on tenofovir and 20 (26%) were on zidovudine based regimens. We did not differentiate whether symptoms were as a result of the disease or the side-effects of treatment as the goal is to improve health outcomes of patients on ART while minimizing adverse events. The majority of the patients reported no skin disorder (96.2%) (**Table 5**). Where these were reported, the most common skin disorders were skin pigmentation and wounds.

**Table 5:** Comparison of the prevalence of symptoms across the regimens.

Type of symptom	Tenofovir (*n*, %)	Zidovudine (*n*, %)	Total (*n*, %)	*p*-Value
**Presence of any symptom**				
Missing	2 (0.7)	3 (1.5)	5 (1.0)	**0.005**
No	241 (80.6)	178 (90.4)	419 (84.5)	
Yes	58 (19.4)	19 (9.6)	77 (15.5)	
Pain	17 (5.7)	3 (1.5)	20 (4.0)	**0.015**
Weight	12 (3.99)	2 (1)	14 (2.8)	**0.038**
**Skin disorder**				
Has a Skin disorder	11 (3.7)	6 (3.0)	17 (3.4)	**0.609**
No skin disorder	288 (95.7)	191 (95.5)	484 (95.6)	
Missing data	2 (0.7)	3 (1.5)	5 (1.0)	
**Type of kind disorder**				
Skin pigmentation	2 (0.7)	1 (0.5)		
Wound	1 (0.3)	2 (1)		
Skin rash	1 (0.3)	1 (0.5)		
Itching	2 (0.7)	1 (0.5)		
Ear peeling	1 (0.3)	0 (0)		
Pimples	1 (0.3)	0 (0)		
Mouth sours	1 (0.3)	0 (0)		
Wound in private part	1 (0.3)	0 (0)		
Herpes	1 (0.3)	0 (0)		
Unspecified skin problem	0 (0.3)	1 (0.5)		
**Psychological problems**				
Emotional	1 (0.3)	1 (0.5)	2 (0.4)	0.360
Loss of concentration and fatigue	1 (0.3)	0 (0.0)	1 (0.2)	
**Liver problem**				
Yellow eyes	1 (0.3)	0 (0.0)	1 (0.2)	0.216
Yellow eyes and swollen legs	2 (0.7)	0 (0.0)	2 (0.4)	
**Musculoskeletal**				
Limping	2 (0.7)	0 (0.0)	2 (0.4)	0.476
Cannot walk	0 (0.0)	1 (0.5)	1 (0.2)	
Change in physical structure	1 (0.3)	0 (0.0)	1 (0.2)	
**Others**				
Tiredness	1 (0.3)	1 (0.5)	2 (0.4)	0.640
Bad breath	0 (0.0)	1 (0.5)	1 (0.2)	0.432
Stroke	1 (0.3)	0 (0.0)	1 (0.2)	0.57
Blindness	0 (0.0)	1 (0.5)	1 (0.2)	0.432
Colds	5 (1.7)	0 (0.0)	5 (1.0)	0.061
Eye problem	1 (0.3)	0 (0.0)	1 (0.2)	0.57
Swollen legs	1 (0.3)	0 (0.0)	1 (0.2)	0.57
Swollen neck	1 (0.3)	0 (0.0)	1 (0.2)	0.57

*NB: Bold under p-value denotes statistical significance.*

There was a statistically significant difference in the prevalence of pain- and weight-related symptoms in patients on zidovudine versus tenofovir based regimens (*p* = 0.015 and 0.038), respectively. The most common type of disorder among all the participants was pain, at 4%, with patients on tenofovir having a higher prevalence of pain (5.7%) (**Table 5**). The sources of pain were backache (*n* = 8; 1.6%), headache (*n* = 4; 0.8%), chest pain (*n* = 3; 0.6%), general body pain (*n* = 3; 0.6%), as well as breast and leg pain (*n* = 2; 0.4%).

The second most common disorder was weight-related changes at 2.8% of all participants in the study. Nearly 4% of participants on tenofovir had a weight-related problem as opposed to only 1% of participants on zidovudine, which was statistically significant (*p* = 0.038; **Table 5**). The most common weight-related problem was weight loss (1.8%). With regard to skin problems, three patients had skin pigmentation, two patients had skin rash, three had itching, and one patient each had ear peeling, pimples, oral sores, genital wounds, and herpes. Three had unspecified skin problems. Only one patient complained of each of the following symptoms: bad breath, blindness, swollen legs, swollen neck, and eye problems. Two patients reported chronic fatigue (0.4%) and 5 (1%) had cold-like symptoms.

### Risk Factors for Any Symptom of Disease or Side-Effects

The risk factors for presence of any symptom were being on tenofovir as well as second line regimens, which remained statistically significant when adjusting for confounding (**Table 6**).

**Table 6:** Risk factors for any symptom of disease or side-effects.

Variable	Crude OR (95% CI)	*p*-Value	Adjusted OR (95% CI)	*p*-Value
**Socio-demographic**				
Age	0.98 (0.959,1.010)	0.239	–	
Gender	1.37 (0.782,2.389)	0.272	–	
Married	0.813 (0.499,1.322)	0.404	–	
Region	0.998 (0.886,1.124)	0.976	–	
Had disclosed status	1.024 (0.562,1.864)	0.939	–	
Education level	1.01 (0.739,1.390)	0.933	–	
Regular source of income	0.990 (0.543,1.806)	0.974	–	
**Baseline medical characteristics**				
HIV duration	1.008 (0.956,1.063)	0.774	–	
ART duration	0.981 (0.916,1.051)	0.586	–	
Missed drug intake	1.345 (0.811,2.231)	0.251	–	
Recent CD4^+^ Count	0.999 (0.999,1.000)	0.276	–	
Recent viral load	1.0 (0.999,1.000)	0.446	–	
**Drug-related factors**				
Zidovudine	0.480 (0.278,0.827)	**0.008**	–	
Tenofovir	2.084 (1.209,3.595)	**0.008**	2.385 (1.356, 4.194)	**0.003**
Nevirapine	0.540 (0.300,0.971)	**0.040**	–	
Efavirenz	1.130 (0.685,1.864)	0.632	–	
Lopinavir/ritonavir	2.520 (1.188,5.341)	**0.016**	–	
Atazanavir/ritonavir	1.570 (0.320,7.701)	0.579	–	
First line regimen	0.421 (0.210,0.843)	**0.015**	–	
Second line regimen	2.376 (1.187,4.757)	**0.015**	2.977 (1.443, 6.144)	**0.003**
Regiment	1.244 (1.090,1.419)	**0.001**	–	

*NB: Bold under *p*-value denotes statistical significance.*

### Comparison of Physical Health Summary Scores of Patients on Tenofovir and Zidovudine

**FIGURE 1:**
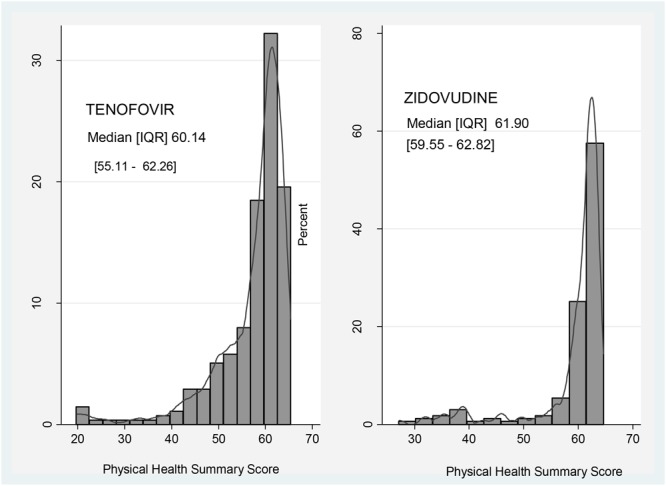
Comparison of Physical Health Summary Score between zidovudine and tenofovir based regimens.

Participants in the zidovudine based arm had a higher median score regarding physical activity (61.90; IQR: 59.55–62.82) compared to those on the tenofovir based regimens (60.14; IQR: 55.11–62.26) (**Figure 1**).

A regular source of income was associated with a 2.62 unit [95% CI (0.456, 4.778)] increase in the PHSS. Being on a zidovudine based regimen also improved the PHSS by 1.33 units [95% CI (0.002, 2.66)] (**Table 7**).

**Table 7:** Regression analysis of determinants of Physical Health Summary Score (PHSS).

Variable	Crude beta coefficient (95% CI)	*p*-Value	Adjusted beta coefficient (95% CI)	*p*-Value
ART regimen (TDF vs. AZT)	2.04 (0.58,3.50)	**0.006**	–	
Sex	–2.04 (–3.46, –0.62)	**0.005**	–1.986 (–3.460,–0.511)	**0.008**
Age	–0.05 (–0.13,0.03)	0.223	–	
Age above 40	–1.46 (–2.83,–0.08)	**0.038**	–1.782 (–3.202,–0.361)	**0.014**
Religious belief	2.62 (–4.78,10.03)	0.487	–	
Education level	0.55 (–0.47,1.56)	0.294	–	
Status disclosure	1.93 (–0.24,4.10)	0.081	–	
Region	–0.03 (–0.37,0.31)	0.856	–	
Has regular source of income	3.07 (0.75,5.39)	**0.010**	2.617 (0.456,4.778)	**0.018**
Marital status	–0.54 (–1.01,0.07)	**0.025**	–	
**HIV disease**				
HIV duration	0.04 (–0.13,0.21)	0.617	–	
ART duration	0.10 (–0.12,0.31)	0.380	–	
Recent CD4^+^ cells/mm^3^	0.003 (–0.0003,0.006)	0.079	–	
Recent viral load copies/ml	7.99e^-08^ (–5.80e^-07^,7.40e^-07^)	0.811	–	
**ART medications**				
Missed drugs intake	–1.39 (–3.04,0.27)	0.100		
Zidovudine	2.025 (0.564,3.485)	**0.007**	1.329 (0.002,2.657)	**0.050**
Nevirapine	0.48 (–1.037,1.996)	0.534	–	
Efavirenz	0.78 (–0.739,2.298)	0.314	–	
Lopinavir/ritonavir	–4.30 (–8.194,–0.403)	**0.031**	–3.773 (–7.418,–0.129)	**0.042**
Atazanavir/ritonavir	–0.26 (–6.4,5.87)	0.932	–	
First line regimen	3.64 (0.205,7.074)	**0.038**	–	
Second line regimen	–3.64 (–7.074,–0.205)	**0.038**	–	
**Coping mechanism**				
Satisfaction with service provision	–0.89 (–2.08,0.29)	0.140	–	
Unable to cope with HIV	–1.92 (–2.79,–1.05)	**<0.001**	–1.813 (–2.562,–1.064)	**< 0.001**
Substance dependence	–0.22 (–0.75,0.31)	0.411	–	
Joined support group	–0.357 (–2.655,1.941)	0.760	–	
Sharing with others	–0.229 (–2.128,1.669)	0.813	–	
Spiritual support	–0.004 (–2.067,2.058)	0.997	–	
Acceptance of status	0.202 (–2.055,2.460)	0.860	–	
**Has signs and symptoms of illness or adverse reaction**				
Reported any symptom	–6.57 (–9.35,–3.79)	**<0.001**	–5.581 (–8.072,–3.091)	**< 0.001**
Has weight disorder	–4.154 (–9.751,1.444)	0.145	–	
Type of weight disorder	–1.53 (–3.941,0.881)	0.213	–	
Has skin disorder	–1.94 (–6.325,2.446)	0.388	–	
Has pain	–8.802 (–13.994,–3.610)	**0.001**	–	

*NB: Bold under *p*-value denotes statistical significance.*

Having a symptom that compromises HRQoL greatly reduced the PHSS by -5.58 [95% CI (-8.072, -3.091)] units. This association remained statistically significant even after adjusting for confounding for type of regimen, stable income, sex, and age. Patients who complained of pain had a lower PHSS than those who did not [crude beta coefficient -8.802, 95% CI (-13.994, -3.610)]. This finding was statistically significant (*p* = 0.001), though was confounded by the type of regimen and lost significance in the multivariable analysis because participants on TDF-based regimens were more likely to complain of pain. Weight changes had no statistical significant impact on PHSS though we noted a negative association. Other factors that reduced the PHSS were female gender [β = -1.986; 95% CI (-3.460, -0.511)], age above 40 [β = -1.782; 95% CI (-3.202, -0.361)], inability to cope with HIV [β = -1.813; 95% CI (-2.562, -0.064)], and being on lopinavir/ritonavir (β = -3.773; 95% CI (-7.418, -0.129)] (**Table 7**) .

### Determinants and Predictors of the Summary Scores (PHSS and MHSS)

**FIGURE 2:**
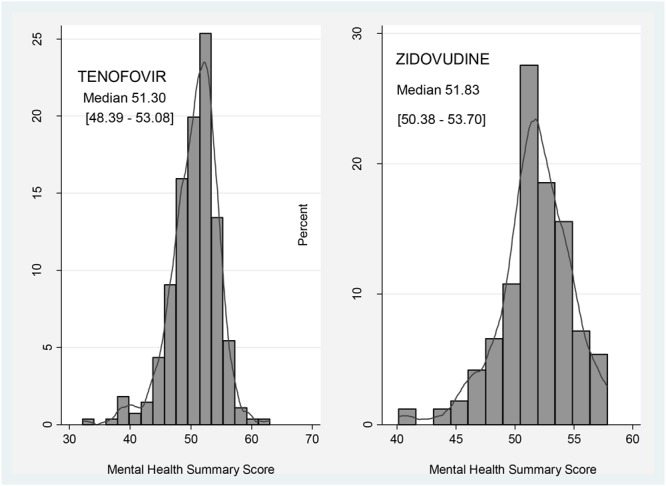
Comparison of Mental Health Summary Score between participants on zidovudine and tenofovir based regimens.

Participants on zidovudine based regimens had a higher median MHSS (51.83; IQR: 50.38–53.70) compared to 51.30 (IQR: 48.39–53.08) of those on the tenofovir based arm, with **Figure 2** comparing the MHSS of participants on zidovudine versus tenofovir based regimens.

Bivariate regression analysis showed that being on an ART regimen (*p* = 0.001), having any symptom that compromises HRQoL (*p* = 0.001), satisfaction with service provision (*p* = 0.004), inability to cope with HIV (*p* < 0.001), and the availability of recent viral load data (*p* < 0.001) all affected the HRQoL of patients (**Table 8**). The presence of any symptoms that compromised the HRQoL was negatively related to the PHSS [β = -2.0; 95% CI (-3.13, -0.85); *p* < 0.001]. The patient stated inability to cope with HIV was attributed to a reduction in the PHSS by -1.12 units [95% CI (-1.58, -0.66)].

**Table 8:** Bi-variable regression analysis – determinants of Physical Health Summary Score.

Variable	Crude beta Coefficient 95% CI	*p*-Value
Patient regimen	1.12 (0.47,1.76)	**0.001**
Sex	–0.58 (–1.28,0.13)	0.111
Age	0.03 (–0.01,0.07)	0.148
Religious belief	–0.24 (–3.35,2.87)	0.877
Education level	–0.04 (–0.47,0.39)	0.859
Status disclosure	0.4 (–0.46,1.26)	0.360
Region	–0.01 (–0.17,0.15)	0.895
Has regular source of income	0.42 (–0.58,1.43)	0.410
Marital status	–0.16 (–0.39,0.06)	0.159
Married	–	–
HIV duration	–0.02 (–0.10,0.06)	0.616
ART duration	–0.04 (–0.14,0.06)	0.467
Any symptom compromising HRQoL	–2.0 (–3.13,–0.85)	**0.001**
Satisfaction with service provision	–0.89 (–1.49,–0.29)	**0.004**
Inability to cope with HIV	–1.12 (–1.58,–0.66)	**<0.001**
Missed drugs intake	–0.26 (–1.0,0.48)	0.486
Alcoholism/substance dependence	0.075 (–0.16,0.31)	0.528
Recent CD4^+^ cells/mm^3^	0.001 (–0.001,0.002)	0.308
Recent viral load copies/ml	2.93e^-07^(1.60e^-07^,4.24e^-07^)	**<0.001**
Zidovudine	1.063 (0.417,1.710)	**0.001**
Nevirapine	0.394 (–0.306,1.093)	0.269
Efavirenz	–0.121 (–0.793,0.552)	0.725
Lopinavir/ritonavir	–0.739 (–1.971,0.493)	0.239
Atazanavir/ritonavir	–4.437 (–2.325,1.451)	0.649
First line	0.701 (–0.384,1.786)	0.205
Second line	–0.701 (–1.786,0.384)	0.205
Type of skin disorder	–0.264 (–0.557,0.030)	0.078
Type of weight disorder	–1.401 (–2.152,–0.650)	**<0.001**
Type of pain	–1.144 (–1.721,–0.568)	**<0.001**
Regimen	–0.261 (–0.410,–0.111)	**0.001**
Has pain	–3.77 (–6.481,–1.052)	**0.007**
Has weight problems	–2.76 (–4.126,–1.39)	**<0.001**
Has skin problem	–1.087 (–3.499,1.324)	0.376

*NB: Bold under *p*-value denotes statistical significance.*

Pain and weight changes had a negative significant effect on the MHSS as presented in **Table 9**. However, the effect of weight changes lost significance on multivariable analysis. After adjusting for confounding for the duration of illness and the type of regimen, the influence of any symptom of an adverse reaction remained significant (**Table 10**).

**Table 9:** Bi-variable regression analysis – variables associated with the Mental Health Summary Score.

Variable	Crude beta Coefficient (95% CI)	*p*-Value
Sex	–0.58 (–1.28,0.13)	0.111
Age	0.03 (–0.01,0.07)	0.148
Education level	–0.04 (–0.47,0.39)	0.859
Region	–0.01 (–0.17,0.15)	0.895
Marital status	–0.16 (–0.39,0.06)	0.159
Has regular source of income	0.42 (–0.58,1.43)	0.410
**HIV Disease**		
HIV duration	–0.02 (–0.10,0.06)	0.616
ART duration	–0.04 (–0.14,0.06)	0.467
Recent CD4^+^ cells/mm^3^	0.001 (–0.001,0.002)	0.308
Recent viral load copies/ml	2.93e^-07^(1.60e^-07^,4.24e^-07^)	**<0.001**
**ART medications**		
Regimen (TDF vs. AZT)	–0.261 (–0.410,–0.111)	**0.001**
Nevirapine	0.394 (–0.306,1.093)	0.269
Efavirenz	–0.121 (–0.793,0.552)	0.725
Lopinavir/ritonavir	–0.739 (–1.971,0.493)	0.239
Atazanavir/ritonavir	–4.437 (–2.325,1.451)	0.649
Second line regimen	–0.701 (–1.786,0.384)	0.205
Missed drugs intake	–0.26 (–1.0,0.48)	0.486
**Has signs and symptoms of illness or adverse reaction**		
Reported any symptoms/signs	–2.0 (–3.13,–0.85)	<0.001
Has skin problem	–1.087 (–3.499,1.324)	0.376
Type of skin disorder	–0.264 (–0.557,0.030)	0.078
Has weight problems	–2.76 (–4.126,–1.39)	**<0.001**
Type of weight disorder	–1.401 (–2.152,–0.650)	**<0.001**
Has pain	–3.77(–6.481,–1.052)	**0.007**
Type of pain disorder	–1.144 (–1.721,–0.568)	**<0.001**
Inability to cope with HIV	–1.12 (–1.58,–0.66)	**<0.001**
**Ability to cope and coping mechanisms**		
Joined support group	–0.029 (–0.951,0.892)	0.950
Sharing with others	0.297 (–0.367,0.961)	0.380
Spiritual support	–0.176 (–0.927,0.575)	0.645
Acceptance of status	0.250 (–0.933,1.434)	0.678
Status disclosure	0.4 (–0.46,1.26)	0.360
Substance dependence	0.075 (–0.16,0.31)	0.528

**Table 10:** Parsimonious models of the Mental Health Summary Score of study participants.

Variable	Most parsimonious model	Parsimonious model in which regimen is replaced by AZT	Model adjusted for duration of ART use
Stated inability to cope with HIV	–0.999 (–1.411,–0.588)	–0.995 (–1.407,–0.582)	–1.029 (–1.441,–0.617)
Absence of pain	0.413 (0.152,0.674)	0.413 (0.152,0.674)	0.414 (0.154,0.675)
Presence of any symptom of illness	–1.240 (–2.253,–0.226)	–1.28 (–2.293,–0.266)	–1.238 (–2.246,–0.230)
Types of ART Regimen	–0.187 (–0.330,–0.045)	–	–0.205 (–0.346,–0.063)
If patient was on AZT	–	0.738 (0.127,1.349)	–
Duration of ART use	–	–	–0.081 (–0.179,0.017)

Overall, the presence of any symptom reduced the MHSS by 1.24 units [95% CI (-2.253, -0.226)] before adjusting for confounders (**Table 10**). This association remained significant even after adjusting for confounding by duration of ART use. The absence of pain score increased the MHSS by 0.413 units [95% CI (0.152, 0.674)] and this association was not confounded by the duration of therapy and being on zidovudine. Being on zidovudine based regimens was also associated with improved mental health with β = 0.738 [95% CI (0.127, 1.349)].

## Discussion

Patients on zidovudine based regimens appeared to have a better HRQoL score compared to those on tenofovir based regimens aided by less adverse events and side-effects. The risk factors for the presence of adverse symptoms and side-effects were being on tenofovir or second line regimens. We were not aware of studies that have directly compared the HRQoL of patients on the two regimens, although studies have compared other outcomes and overall cost-effectiveness between the two regimens (von Wyl et al., 2012; Velen et al., 2013; Ayele et al., 2017a,b). Consequently, we believe this is probably the first study in this area, and certainly we believe within sub-Saharan Africa.

However, our findings appear to contradict a number of other studies that have assessed the two regimens in terms of their safety and clinical outcomes, which have reported better outcomes and tolerability with tenofovir. A systematic review and meta-analysis of the efficacy and tolerability of the tenofovir based regimen (TDF/3TC/EFV) and the zidovudine based regimen (AZT/3TC/EFV) by Dadi et al. reported better outcomes for TDF/3TC/EFV when compared to AZT/3TC/EFV (Dadi et al., 2017). Velen et al. also reported better performance with tenofovir based regimens as opposed to zidovudine regimens (Velen et al., 2013). We are not sure of the reasons behind these differences; however, we will be exploring this further in future studies. Our postulation is that different sample sizes among the studies could have played a role.

There was a positive correlation between a regular source of income and improved PHSS, which is similar to a number of studies illustrating that HRQoL is positively related to income (Mafirakureva et al., 2016). As a result, a range of efforts should be put in programs in order to improve the QoL in HIV patients (Van Tam et al., 2012; Vagiri et al., 2014). This is in addition to instigating treatments that can improve symptoms without appreciable toxicity. This is because the side-effects of treatment as well as adverse symptoms greatly reduce both PHSS and the MHSS, and hence HRQoL.

The overall prevalence of adverse symptoms and side-effects in our study (**Tables 5, 6**) was low (15.5%), and much lower that a previous study conducted at Kiambu sub-county among persons living with HIV, reporting a 65.2% prevalence of symptoms suggestive of ADRs (Nderitu et al., 2013). This is important as the side-effects of therapy can greatly reduce adherence to ART even if they are provided free-of-charge to patients (Nderitu et al., 2013). The prevalence of any symptoms in our study was 15.5%, similar to that previously seen among HIV patients with opportunistic infections in the same hospital (Awino, 2008).

Participants on the tenofovir based arm had a higher prevalence of symptoms or side-effects and consequently had a lower HRQoL compared to those on zidovudine. Previous pharmacovigilance studies on ADRs, conducted among patients in developing countries, also reported side-effects associated with tenofovir (Subbaraman et al., 2007; Nemaura et al., 2013; Waheed et al., 2015). The risk factors for the presence of symptoms or side-effects in this study were being on tenofovir or second line regimens. This is important for guiding future treatment strategies in sub-Saharan countries such as Kenya.

Among the participants, 15.5% reported a symptom which ranged from pain, dermatological reactions, and confusion. Regression analysis confirmed that presence of any symptom had a negative effect on both the PHSS and MHSS. Following regression analysis, this reduced the physical health of patients by -6.05 (-8.61, -3.49) units while the mental health was reduced by -1.85 (-2.72, -0.98) units. This concurs with a number of studies that have demonstrated negative effects of signs or symptoms of illness on QoL (Dadi et al., 2017; Pasquau et al., 2018). For instance, a study on the effects on ART in the public sector in South Africa by Wouters et al. highlighted the need for clinicians to be vigilant about the adverse effects of treatment since these can have severe effects on both physical and emotional QoL (Wouters et al., 2009).

We are aware this study had limitations. First, the subjective nature of the questionnaire implies that some of the claims by the patients could not be verified; however, arguably, using patient’s self-reported HRQoL could be considered a strength since patient HRQoL is a subjective phenomenon and can only be reported directly by the patient, i.e., it cannot be observed and therefore not reported by a clinician or caregiver. We are also aware that the study did not clearly differentiate the symptoms of the disease with the side-effects of the treatment. In addition, by default, the numbers of participants on tenofovir based regimens were greater than those on the zidovudine based regimen in accordance with the Kenyan treatment guidelines. This study was also only conducted at one city and center in Kenya. Having said this, we believe the study had a comparatively large sample size making it reasonably representative of the patient population with HIV in Kenya. In addition, KNH is the tertiary center where patients with HIV in Kenya come to be treated.

The study also highlighted the need to evaluate the patients rating of their own health, with the key components of health being physical and mental health as perceived by patients, which were evaluated in this study. The use of the disease specific MOS-HIV questionnaire enables the findings to be compared to other studies where similar questionnaires are used. Consequently, we believe our findings are robust providing direction for the future.

## Conclusion

Patients on zidovudine had better PHSS and MHSS compared to those on tenofovir based regimens. However, we believe additional studies are required to confirm these findings, especially in view of the differences seen in our study versus others regarding the side-effects of treatment with these two treatments, before definitive guidance can be given on which regimens to preferentially use. These are considerations for the future, and we will be progressing this research in our hospital.

## Author Contributions

JE, FO, SO, and GO designed the concept of the study, undertook the analysis, and with JE led the interview team. AK, JM, and BG also helped with the analysis. JE, SO, JM, MO, and BG developed the first draft of the manuscript. All the authors contributed to the successive refinements and approved the final version before submission.

## Conflict of Interest Statement

The authors declare that the research was conducted in the absence of any commercial or financial relationships that could be construed as a potential conflict of interest.
